# Adipokines as Cardioprotective Factors: BAT Steps Up to the Plate

**DOI:** 10.3390/biomedicines13030710

**Published:** 2025-03-13

**Authors:** Keely McLeod, Victoria Datta, Scott Fuller

**Affiliations:** 1 School of Kinesiology, University of Louisiana at Lafayette, Lafayette, LA 70506, USA; keely.mcleod1@louisiana.edu (K.M.); victoria.datta@louisiana.edu (V.D.); 2 Pennington Biomedical Research Center, Louisiana State University System, Baton Rouge, LA 70808, USA

**Keywords:** adipokines, brown adipose tissue, batokines, cardiovascular disease, metabolic syndrome, insulin resistance, obesity, type 2 diabetes, atherosclerosis, hypertension, neuregulin-4, NRG4, FGF21, 12,13-diHOME, microRNA

## Abstract

Cardiovascular disease is the leading cause of death throughout most of the industrialized world. Metabolic syndrome (MetS) and its associated pathologies are underlying factors in the etiology of cardiovascular disease, as well as a plethora of other maladies which cause excess morbidity and mortality. Adipose tissue (AT) has come to be regarded as a bona fide endocrine organ which secretes specific molecular entities constituting part of a complex web of inter-organ crosstalk that functions as a key determinant of whole-body metabolic phenotype. Brown adipose tissue (BAT) has classically been regarded as a thermogenic tissue exerting its metabolic effects primarily through its capacity to oxidize substrates decoupled from ATP resynthesis, thereby resulting in increased energy expenditure (EE) and heat production. However, in recent years, BAT has begun to receive attention as a secretory organ in its own right. The molecules secreted specifically by BAT have been termed “batokines”, and currently available evidence supports the notion that batokines exert favorable metabolic effects on multiple organ systems. While maintenance of healthy body composition by conferring resistance to excessive adiposity is a rather obvious mechanism by which BAT operates via increased EE, effects on critical organs such as the heart remain unclear. This narrative review focuses on four types of batokines (FGF21, neuregulin 4, 12,13-diHOME, and BAT-derived microRNAs) for which evidence of modulation of cardiovascular function exists in the context of pathological states such as hypertension, atherosclerosis, and ischemia/reperfusion injury. Given the overwhelming burden of cardiometabolic disease, further study of the functions of BAT and its secretome is warranted and will intensify in the future.

## 1. Introduction

Adipose tissue (AT) has long been recognized as a regulator of nutrient status given its capacity to store excess dietary substrate as triacylglycerols (TGs) in the fed state and export fatty acids and glycerol during fasting [1,2]. However, the discovery of secretory factors specifically derived from adipocytes in the mid-1990s has led to an appreciation of adipose tissue beyond its function solely as a storage site for excess dietary substrate [3,4]. Indeed, the roughly concurrent discoveries of leptin, adiponectin, TNF-α, adipsin, and other adipocyte-derived factors termed “adipokines” have given rise to a surge in interest in adipose tissue as a bona fide endocrine organ situated at the nexus of energy homeostasis [5].

The recognition that adipocytes play an indispensable role in metabolic function also provided an impetus for the development of pharmacologic agents targeting adipose tissue specifically in efforts to improve insulin sensitivity and blood glucose control [6,7] as part of the continuing struggle to combat type 2 diabetes worldwide. The anti-diabetic drugs targeting adipose tissue exert their effects primarily by modulating the activity of the transcription factor PPAR-γ, which is the master transcriptional regulator of adipogenesis [8]. These PPAR-γ agonists constitute thiazolidinediones (TZDs), which have potent insulin-sensitizing effects but are known to result in weight gain and can also have adverse effects on cardiac function [9]. Given that cardiovascular disease (CVD) is one of the leading causes of death globally [10,11] and that obesity-related metabolic dysfunction is a critically important contributor to CVD [12], there has never been a more urgent time to increase understanding of the complex relationships between adipose tissue of all types and cardiovascular function [13].

**Figure 1:**
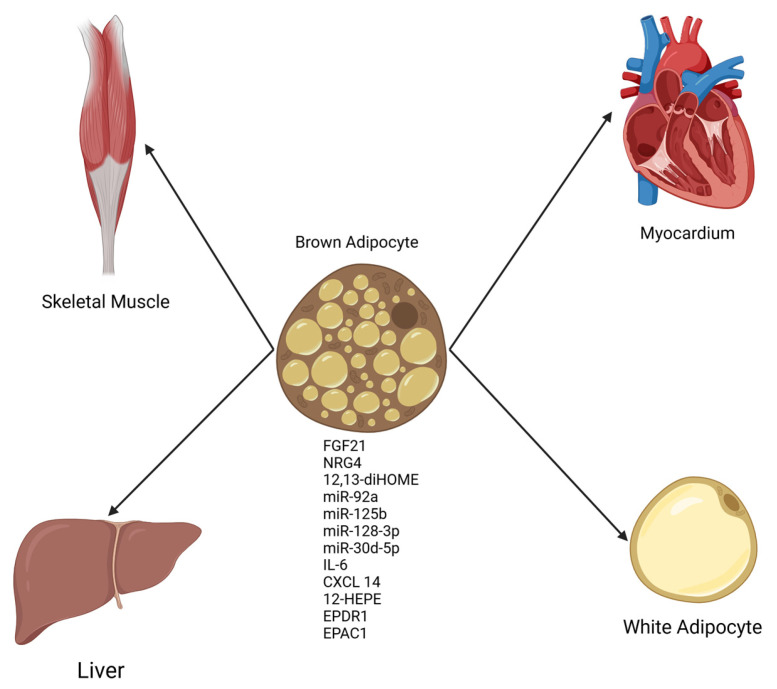
General schematic of factors secreted from BAT (batokines) with effects on multiple organs. Batokines appear to be uniformly beneficial in organ systems studied thus far [28]. Created in BioRender.com.

The discovery that functional brown adipose tissue (BAT) exists in adult humans [14] led to a groundswell of research interest in this unique tissue. BAT had been long recognized as a physiological defense against cold temperatures in small mammals and human infants given its capacity to generate heat by dissipating the normally tight coupling between substrate oxidation and ATP synthesis [15]. However, in recent years, an appreciation of BAT as a tissue with secretory function (see Figure 1, which is provided as a general reference aid to the reader on a wide range of molecules secreted from BAT) has developed in parallel with our current understanding of WAT as an endocrine organ with potent effects on whole-body metabolic function [5,16,17]. Notably, several studies have indicated that diminished BAT activity and non-functional BAT are contributors to obesity and cardiometabolic syndrome [18,19,20]. Conversely, expansions in BAT mass and increases in the circulating levels of BAT-derived secreted molecules (batokines) have been demonstrated by a growing body of literature to beneficially affect key physiological parameters such as glucose homeostasis, insulin sensitivity, and total energy expenditure, all of which confer resistance to the development of obesity-related metabolic dysfunction [21,22,23].

While the discovery of functional BAT in adult humans constituted a major leap forward in our understanding of adipose tissue in general as a nexus of metabolic control in concert with the actions of WAT, the recognition of BAT-derived secreted factors as modulators of cardiovascular function is a newly emerging field [24,25,26,27]. Recent studies have identified several batokines that modulate the heart in a generally favorable manner; these include FGF21, neuregulin 4 (NRG4), 12,13-diHOME, and microRNAs specifically derived from BAT [28]. This review will focus specifically on these four types of batokines for which experimental evidence exists demonstrating a heretofore relatively unknown function of BAT as a tissue acting in a cardioprotective manner and will aim to concisely summarize the state of the field in understanding the mechanisms by which these BAT-derived humoral factors could act advantageously in combating CVD. Specifically, we aim to highlight the insights gained mainly from recent preclinical studies on the batokines FGF21, NRG4, 12,13-diHOME, and miRNAs secreted from BAT on how these factors favorably alter cardiovascular function by enhancing contractile performance and myocardial calcium cycling, inhibiting cardiac fibrosis and cardiomyocyte apoptosis in response to injury, and reducing oxidative stress and inflammation, as examples. The new recognition of BAT as a cardioprotective tissue is certain to stimulate vigorous investigation in the future, with a view towards targeting BAT expansion and enhanced secretory function as a potential therapeutic strategy in combating cardiometabolic disease.

## 2. Batokines Affecting Cardiac Function

### 2.1. FGF21

Fibroblast growth factor 21 (FGF21) is expressed primarily in the liver, although recent evidence indicates that this peptide is also produced by cardiomyocytes and BAT [29,30,31]. FGF21 has received intense attention in the scientific literature in the context of metabolic syndrome due to the generally beneficial effects observed on systemic parameters such as glucose homeostasis, insulin sensitivity, and systemic inflammation [32]. Moreover, FGF21 is involved in the adaptive responses to prolonged fasting and ketogenic diets, which include enhanced insulin sensitivity and cognitive effects [33,34,35]. FGF21 is therefore viewed as a key integrator of a diverse range of physiological responses to states of energy restriction, many of which underlie desirable alterations in metabolism that confer resistance to clinical manifestations of metabolic syndrome.

While the centrality of FGF21 as a factor underlying physiological adaptations to challenges in whole-body energy status has been widely recognized, appreciation of the role of FGF21 as an important mediator of many of the beneficial effects of increased activity of BAT thermogenesis has only recently emerged. This is particularly relevant in terms of cardiac function since the discovery that the heart both produces FGF21 in cardiomyocytes and is in turn stimulated by FGF21 derived from distant tissues; therefore, FGF21 exerts endocrine, paracrine, and intracrine effects on the cardiovascular system in a complex system of inter-organ crosstalk [36,37,38,39,40,41]. As early as 2013, transgenic mice studies demonstrated that FGF21 KO mice suffered maladaptive cardiac hypertrophy and dilatation in response to isoproterenol challenge and that the maladaptive responses were reversed by treatment with exogenous FGF21 [41]. More recent studies indicate that BAT is a source of FGF21 [30] and that FGF21 derived specifically from BAT exerts a protective effect on the heart by acting as a preventative against deleterious cardiac remodeling in response to hypertension in mice [31]. Experiments from this study revealed a crucial role for FGF21 from interscapular BAT in attenuating hypertensive cardiac remodeling, which was intertwined with BAT adenosine A_2A_ receptor signaling, which was obligatory in mediating FGF21 protection against pathological hypertensive cardiac remodeling. Moreover, this study showed that the beneficial cardiac effects of BAT-derived FGF21 were dependent on AMPK/PGC1α pathway activation, which was necessary for the induction of FGF21 secretion from iBAT. The conclusions of this study were consolidated by the finding that administration of recombinant FGF21 in iBAT-depleted mice improved cardiac remodeling and that intact BAT-specific A_2A_ receptor signaling was required to diminish cardiac damage in hypertensive mice [31]. Importantly, BAT-derived FGF21 has also recently been demonstrated as a cardioprotective factor in C57BL/6J mice subjected to myocardial ischemia/reperfusion injury (I/R), which is a major causative factor in myocardial damage resulting from infarction [42]. Data from a study by Ding et al. provided evidence from in vitro experiments that the protective effect of BAT-derived FGF21 in the context of dexmedetomidine administration was modulated by the Keap/Nrf2 pathway, whereby FGF21 stimulation of Nrf2 signaling attenuated the oxidative stress and inflammation typically observed in myocardial I/R injury [43]. Table 1 provides a brief summary of studies cited in the text of this review.

### 2.2. NRG4

Neuregulin 4 (NRG4), a lesser-known but emerging batokine secreted by brown adipose tissue (BAT), has received significant attention for its key role in cardioprotection through its multifaceted involvement in energy metabolism and homeostasis [44]. Initially detected in the pancreas [45], NRG4 belongs to the epidermal growth factor family and preferentially binds to the receptor tyrosine kinase ErbB4, predominantly expressed in adipose tissue [46]. Recent studies (see Table 2 for a summary) provide evidence for NRG4’s potential cardioprotective properties, indicating its crucial role in mitigating the adverse impacts of obesity, insulin resistance, and metabolic dysfunction on heart health [47]. Insulin resistance, defined by disrupted insulin-mediated glucose metabolism, is recognized as an early indicator of cardiovascular disease [48]. Strong evidence supports the role of BAT in alleviating insulin resistance [49], with NRG4 playing a pivotal role in enhancing glucose metabolism and reducing inflammation in metabolic tissues [44]. Hyperinsulinemic–euglycemic clamp studies have demonstrated that NRG4 increases glucose metabolism in peripheral tissues, significantly improving systemic insulin sensitivity [50]. Furthermore, NRG4 reduces chronic inflammation in white adipose tissue by decreasing macrophage accumulation in high-fat-diet-induced obese mice, further supporting its role in improving metabolic health [51].

Obesity significantly contributes to cardiovascular disease risk by dysregulating lipid metabolism, leading to the ectopic accumulation of lipids in various tissues and provoking cardiovascular complications [52,53]. Cai et al. demonstrate that mRNA expression of NRG4 is reduced in adipose tissue in human models of obesity [54], while Chen et al. observe similar findings in mouse models [50]. Activating NRG4 in adipocytes has been shown to enhance adipose tissue angiogenesis, suggesting a pro-angiogenic role for NRG4 [55]. Furthermore, overexpressing NRG4 through hydrodynamic gene delivery significantly reduces diet-induced obesity in mice [51], demonstrating NRG4’s therapeutic potential and contribution to cardiovascular disease reduction.

NRG4’s role in metabolic regulation forms the foundation of its external cardioprotective effects; however, it also exerts a direct, internal cardioprotective influence. In vivo and in vitro studies have revealed NRG4’s activity in restoring cardiac function and attenuating pathological remodeling [56]. Specifically, in an isoproterenol-induced cardiac remodeling study, NRG4 treatment was shown to significantly restore cardiac function, mitigate pathological hypertrophy, and suppress myocardial fibrosis [56]. These therapeutic effects were mechanistically linked to its modulation of inflammatory responses and apoptosis via activation of the AMPK/NF-κB signaling pathway [56]. The AMPK/NF-κB pathway primarily suppresses inflammation and apoptosis, thereby preventing tissue damage and reducing pathological remodeling of cardiac tissue [56]. Complementary to this, the AMPK/Nrf2 pathway plays a pivotal role in regulating oxidative stress and ferroptosis by enhancing antioxidant defenses [57]. In a diabetic myocardial injury model, NRG4 alleviated high-glucose-induced ferroptosis in cardiomyocytes by activating the AMPK/Nrf2 signaling pathway [57]. Notably, inhibition of this pathway diminished NRG4’s beneficial effects, emphasizing the importance of AMPK signaling in cardiovascular protection. Both AMPK-mediated pathways have been linked to the amelioration of cardiovascular disease.

At the vascular level, NRG4 has been shown to prevent endothelial dysfunction and vascular inflammation [58]. In a mouse model, BAT-specific NRG4 deficiency exacerbated atherosclerosis, whereas restoring NRG4 levels reversed these effects, demonstrating its crucial role in maintaining vascular health [58]. Beyond experimental models, clinical studies have identified profound serum NRG4 levels as a promising biomarker for cardiovascular protection [59]. Lower serum NRG4 has been associated with early detection of vascular abnormalities, such as increased carotid intima media thickness and atherosclerotic plaque, particularly in individuals at high risk of subclinical cardiovascular disease [59,60]. Reduced NRG4 levels have also been observed in patients with coronary artery disease [61]. In concordance with these findings, a recent Mendelian randomization study found that elevated serum NRG4 plays a protective role in atherosclerosis, mediating lowered LDL-C levels linked to a reduced risk of peripheral atherosclerosis [62]. By enhancing insulin sensitivity, regulating lipid metabolism, and protecting against cardiac remodeling, NRG4 represents a promising therapeutic target in addressing heart disease, particularly in populations affected by obesity and type 2 diabetes [47,63]. While NRG4 has demonstrated cardioprotective effects largely in animal studies, its exact role in the cardiovascular system is not yet fully understood, and further investigation is needed to further clarify its complete potential.

We take care to note that the preponderance of the studies performed thus far examining the relationships between NRG4 and cardiac function, particularly at the mechanistic level, have been performed in rodents and are thus preclinical. Moreover, these studies have generally used pharmacologic or genetic manipulations in NRG4 expression and the results therefore might diverge from more physiological methods of stimulating NRG4 from BAT, such as cold exposure or exercise. Also, the comparatively few studies investigating the relationships between NRG4 and cardiac function performed in humans have examined correlations between pathological states such as obesity, where NRG4 expression is diminished, and observed negative cardiometabolic health outcomes in such states. Insofar as these results from human studies are correlational rather than experimental, they offer limited insight into whether manipulation of NRG4 is directly beneficial for human cardiac health. Controlled trials with modalities such as exercise and short-term cold exposure, which are known physiological stimulators of the BAT secretome, would provide valuable evidence indicating the extent to which NRG4 might favorably alter cardiac function and the duration and intensity of interventions necessary to evoke beneficial changes.

**Table 2:** Summary of studies examining NRG4 and cardioprotection.

Year	First Author	Citation	NRG4 Cardioprotective Outcomes (Systemic or Direct)	Model
2017	Chen	[50]	↑ Energy expenditure, ↑ Whole-body glucose metabolism, ↑ β-oxidation, ↑ Glycolysis, ↓ Hepatic steatosis, ↓ Inflammation (eWAT)	C57BL/6J Nrg4 transgenic mice, 10–12 weeks old, obesity induced by HFD (60% kcal fat)
2016	Ma	[51]	↓ Diet-induced weight gain, ↓ Inflammation, ↓ Macrophage infiltration, ↑ BAT thermogenesis, ↑ Insulin sensitivity, ↓ Hepatic steatosis, ↓ Nrg4 mRNA (AT, pre-delivery)	C57BL/6 mice, HFD-induced obesity (60% kcal fat), Nrg4 overexpression via hydrodynamic gene transfer
2016	Cai	[54]	↓ Serum Nrg4, ↑ MetS, ↑ Blood glucose and BP	Obese adults (≥40 years), serum Nrg4 measurement
2018	Nugroho	[55]	↑ Adipose tissue angiogenesis, ↑ WAT vasculature, ↑ Systemic metabolic health, ↓ Adipose hypoxia, ↓ Inflammation, ↑ Glucose homeostasis	C57BL/6 aP2-Nrg4-Tg mice, HFD (35% kcal fat), 16 weeks old, angiogenesis inhibition (SU-5416)
2024	Wei	[56]	↑ Cardiac function, ↓ Cardiac hypertrophy, ↓ Fibrosis, ↓ Cell apoptosis, ↓ Inflammatory factors, ↑ Cardioprotection via AMPK/NF-κB pathway	C57BL/6J mice (8 weeks, ISO-induced cardiac remodeling, Nrg4 supplementation)
2024	Wang	[57]	↓ Myocardial injury, ↓ Oxidative stress, ↓ Ferroptosis, ↑ AMPK/Nrf2 signaling, ↑ Cardiac function, ↓ Cardiac fibrosis, ↑ Mitochondrial integrity	C57BL/6J mice, 8 weeks old, STZ-induced T1D, Nrg4 supplementation
2022	Shi	[58]	↓ Atherosclerosis, ↓ Vascular inflammation, ↓ Endothelial dysfunction, ↓ Leukocyte homing, ↓ Apoptosis, ↓ Inflammation	C57BL/6J mice, 4–6 weeks old, fed WD (41% fat), BAT-specific Nrg4 deletion/restoration. CAS human patients (35–64 years); plasma Nrg4.
2016	Jiang	[60]	↓ Serum Nrg4 levels associated with ↑ CIMT and carotid plaque, ↑ Nrg4 levels associated with ↓ BMI, ↓ Systolic BP, ↓ Total cholesterol	Obese humans (≥40 years, serum Nrg4 measured, CIMT and carotid plaque assessed)
2023	Taheri	[61]	↓ Nrg4 levels in CAD, ↑ BMI, ↑ Waist circumference, ↑ Fasting blood glucose, ↑ Triglyceride–glucose index	CAD human patients (50–65 years), serum Nrg4 measured
2014	Zheng	[62]	↓ Athereosclerosis, ↓ LDL-C levels, ↓ Peripheral atherosclerosis	Mendelian randomization study (1.32 million individuals)

Nrg4, neuregulin four; HFD, high-fat diet; WD, Western diet; eWAT, epididymal white adipose tissue; BAT, brown adipose tissue; MetS, metabolic syndrome; BP, blood pressure; AMPK/NF-κB, adenosine monophosphate-activated protein kinase/nuclear factor kappa B; AMPK/Nrf2, adenosine monophosphate-activated protein kinase/nuclear factor erythroid 2-related factor 2; CIMT, carotid intima media thickness; CAS, carotid atherosclerosis; CAD, coronary artery disease; BMI, body mass index; LDL-C, low-density lipoprotein cholesterol; T1D, type 1 diabetes. ↑ increased, ↓ decreased.

### 2.3. 12,13-diHOME

In 2008, Cao and colleagues identified a novel lipid hormone connecting adipose tissue to organism-wide metabolism; these investigators termed this newly discovered hormone “lipokine” [64]. Recent experiments have provided evidence that a novel lipokine secreted from BAT, 12,13-diHOME (12,13-dihydroxy 9Z-octadecenoic acid), exerts beneficial cardiac effects [65,66]. Interestingly, 12,13-diHOME is increased independently of ambient temperature by a single bout of exercise in humans and mice, with evidence demonstrating that the tissue source of 12,13-diHOME is BAT, as shown by surgical ablation of interscapular BAT negating the exercise-induced increase in 12,13-diHOME [67]. These experiments revealed the mechanistic insight that 12,13-diHOME increases skeletal muscle fatty acid uptake and oxidation via induction of genes involved in fatty acid transport and mitochondrial activity and biogenesis. Other experiments demonstrate that 12,13-diHOME treatment increases mobilization of fatty acid transporters CD36 and FATP1 to the membrane in mature brown adipocytes, thereby increasing uptake of fatty acids and setting the stage for increased fat oxidation [65]. Given that exercise is an indispensable cardioprotective modality, the close linkage between exercise and lipokine secretion from BAT indicates that 12,13-diHOME can be thought of as an “exerkine,” a term referring to humoral factors induced by exercise that confer pleiotropic metabolic benefits [68,69,70]. Please see Table 3 for a summary of studies cited in this review that investigate the cardiometabolic effects of 12,13-diHOME.

From the perspective of specific cardioprotection, 12,13-diHOME has been shown in mice to favorably modulate cardiac function directly via increasing mitochondrial respiration, inducing nitric oxide synthase 1 (NOS1), and increasing cardiomyocyte contractility by NOS1-dependent activation of the ryanodine receptor [66]. These findings were consistent with previous data demonstrating that NOS1 modulation of cardiac contractile function is mediated by calcium cycling via interaction with the ryanodine receptor [71,72]. Moreover, this study provided evidence that 12,13-diHOME is positively correlated with ejection fraction in human patients with heart disease [66]. While previous studies have indicated that 12,13-diHOME could be deleterious to cardiac health, the applicability of these results is vitiated by methodological issues, including the use of ex vivo models and high concentrations of 12,13-diHOME that are toxic to cardiomyocytes [66,73,74,75]. A retrospective study undertaken by Cao and colleagues on T2DM patients with and without acute myocardial infarction (AMI) employed untargeted metabolomics and subsequent validation with ELISA to demonstrate that 12,13-diHOME was elevated in T2DM patients with AMI compared to those who did not suffer AMI [76]. However, this study is retrospective and correlational; therefore, it is difficult to impute a causal role for 12,13-diHOME in AMI from these data alone [76].

In contrast with these apparently adverse associations between 12,13-diHOME and cardiometabolic health outcomes, evidence of beneficial effects of 12,13-diHOME on cardiovascular function was shown in mouse experiments demonstrating a linkage between insulin signaling and differentiation of perivascular progenitor cells (PPCs) into BAT, with consequential increases in BAT mass and 12,13-diHOME secretion, which in turn reduced inflammation and atherosclerosis in mice [77]. These experiments indicated that inhibition of eNOS by L-NAME resulted in a failure of PPCs to differentiate into beige/brown adipocytes, the abrogation of weight loss, and beneficial effects on bioenergetics which were reversed by infusion of 12,13-diHOME, which improved endothelial function and decreased atherosclerosis [77]. These recent results in preclinical studies are encouraging; however, future research in humans with and without diagnosed CVD are needed to more thoroughly understand the effects of 12,13-diHOME on cardiovascular function.

**Table 3:** Summary of studies examining 12,13-diHOME and cardioprotection.

Year	First Author	Citation	12,13-diHOME Cardioprotective Outcomes (Systemic or Direct)	Model
2017	Lynes	[65]	↑ BAT activity, ↑ fat oxidation, ↑ cold tolerance, ↓ serum triglycerides, ↑ fatty acid uptake, ↑ lipid metabolism	C57BL/6J mice, 12 weeks old, cold exposure (4 °C), NE treatment, HFD-induced obesity (60% kcal fat); human study, cold exposure, lipidomic analysis
2021	Pinckard	[66]	↑ Mitochondrial respiration, ↓ cardiac remodeling, ↑ NOS1 activity, ↑ cardiomyocyte contractility, ↑ systolic function, ↑ diastolic function, ↓ 12,13-diHOME in heart disease, ↑ glucose tolerance, ↑ fatty acid uptake, **↑** ejection fraction	C57BL/6 mice (12 weeks old), BAT transplantation; heart failure patients (62–65 years), lipidomics analysis
2018	Stanford	[67]	↑ Baseline 12,13-diHOME in active individuals, ↑ circulating 12,13-diHOME post-exercise, ↑ fatty acid uptake, ↓ RER, ↑ mitochondrial respiration, ↑ fatty acid oxidation	Healthy humans (21–90 years); C57BL/6 mice (10–12 weeks), iBAT removal
2007	Gonzalez	[71]	NOS1 deficiency → ↓ RyR2 S-nitrosylation, ↑ SR Ca^2^^+^ leak, ↓ SR Ca^2^^+^ content, ↑ ventricular arrhythmias, ↑ sudden cardiac death	C57BL/6 (3–6 months), NOS1−/− and NOS3−/−KO, Ca^2^^+^ homeostasis and RyR2 S-nitrosylation analysis
2022	Park	[77]	↑ BAT mass, ↑ 12,13-diHOME secretion, ↓ inflammation, ↓ atherosclerosis, ↑ endothelial function, ↑ insulin signaling, ↑ thermogenesis	C57BL/6J mice (10 weeks old), HFD (60% kcal fat), atherosclerosis induction

12,13-diHOME, 12,13-dihydroxy-9Z-octadecenoic acid; BAT, brown adipose tissue; HFD, high-fat diet; NE, norepinephrine; NOS1, neuronal nitric oxide synthase; NOS3, endothelial nitric oxide synthase; KO, knockout; RyR2, ryanodine receptor 2; SR, sarcoplasmic reticulum; Ca^2^^+^, calcium; iBAT, interscapular brown adipose tissue; RER, respiratory exchange ratio. ↑ increased, ↓ decreased.

### 2.4. BAT-Derived miRNA Affects Cardiac Function

Small extracellular vesicles (sEVs) are secreted from various tissues upon exposure to physiological and pathological stimuli, whereupon they enter the circulatory system and constitute an important biological mechanism by which diverse organ systems communicate with one another [78,79]. Also known as exosomes, sEVs have become acknowledged as playing a major role in regulating inter-organ crosstalk in an expanding body of literature in which experimental evidence in numerous paradigms indicates that sEVs are obligatory in mediating the beneficial effects of interventions such as exercise [78,79,80,81,82]. As our understanding of the pleiotropic benefits of BAT activation expands, it is notable that sEVs are emerging as key mechanistic players mediating the beneficial effects of BAT. This section of the review will summarize the latest findings providing evidence for a specific role of BAT-derived sEVs in cardioprotection, which is a relatively new field of exploration that offers potential in unraveling the mechanisms by which BAT can communicate not only with the heart but with other distant organs as well. Please see Table 4 for a summary of studies discussed in the text of sEVs from BAT and their cardiometabolic effects. 

A seminal set of experiments performed by Zhao and colleagues revealed that a subset of sEVs from BAT exerted potent direct cardioprotective effects of exercise in a model of myocardial infarction/reperfusion (MI/R) injury in mice [81]. Specifically, the findings of these experiments demonstrated that a particular set of miRNAs contained in the sEVs from BAT (miR-125b-5p, miR128-3p, and miR30d-5p) favorably modulated the heart’s response to MI/R injury via suppression of the proapoptotic MAPK pathway [81], thereby promoting cardiomyocyte survival. Mechanistic insights from this study from in vivo proof-of-concept experiments and detailed investigation from in vitro studies investigated the mechanisms by which the miRNA cargo of BAT conferred exercise-induced cardioprotection. The data from these experiments revealed that the main biological mechanisms by which sEVs from BAT advantageously modulated cardiac response to MI/R injury were by inhibition of several genes in the proapoptotic MAPK and caspase pathways, including *Map3k5*, *Map2k7*, and *Map2k4*. The conclusions of the study were consolidated by data indicating that direct delivery of BAT sEVs into hearts or cardiomyocytes inhibited MAPK pathway activation and that this protective effect was abolished by BAT miRNA inhibitors [81]. Collectively, the results of these in vitro and in vivo experiments revealed a novel pathway by which the cardiovascular benefits of aerobic exercise are mediated by activation of the BAT secretome, specifically miRNAs encapsulated by sEVs.

The effect of miRNAs on heart function remains complex and is an emerging field of study. A particular miRNA, miR-92a, has been investigated and reveals a complicated relationship with BAT and cardiac function which has yet to be fully unraveled. The circulating levels of miR-92a are negatively correlated with BAT activity in humans; however, currently it is not possible to definitively conclude whether or not miR-92a is an independent causal factor in cardiac pathology [83,84,85,86]. Chen and colleagues, for example, demonstrated that miR-92a abundance in exosomes was markedly decreased in both humans and mice in conditions with high BAT abundance or activity, such as in mice subjected to cold exposure or cAMP treatment, both of which are known to activate BAT [86]. Plasma miR-92a levels were also shown to be elevated in patients with diabetes with concomitant increased expression of the inflammatory factors NF-κB, MCP-1, ICAM-1, and endothelin-1 [87]. Another study indicated that in a porcine experimental model, inhibition of miR-92a resulted in protection against MI/R injury, with improved ejection fraction, left ventricular end-diastolic pressure, and reduced infarct zone size [84].

While the previously cited studies implicate miR-92a in myocardial damage, drawing definitive conclusions is complicated by a recent study showing that overexpression of miR-92a-5p attenuated myocardial damage in rats with induced diabetic cardiomyopathy [83]. In these rats, the principal mechanism that conferred cardioprotection was a reduction in oxidative stress injury, with reduced apoptosis levels, increased glutathione, reduced malondialdehyde accumulation, and inhibition of MKNK2 expression, which in turn reduced activation of p38MAPK signaling [83]. Taken together, the divergent results of these studies might indicate that a physiological secretion of miR-92a from activated BAT could be cardioprotective, while in a different context, this miRNA could be damaging to the heart. Any conclusions currently remain speculative until more detailed mechanistic experiments can be performed that can clarify the biological conditions under which miR-92a acts beneficially versus adversely.

**Table 4:** Studies examining BAT-derived MiRNA effects on cardiac function.

Year	First Author	Citation	MiRNA Cardioprotective Outcomes	Model
2022	Yu	[83]	↑ MiR-92a results: ↑ Glutathione level, ↓ myocardial oxidative stress, ↓ ROS, ↓ malondialdehyde, ↓ apoptosis, ↓ MAPK signaling	Adult male Sprague Dawley rats; type 2 diabetes induced by high-fat diet (4 weeks) + streptozotocin; miR-92a-2-5p overexpression via adenovirus transfection (in vitro).
2022	Zhao	[81]	↑ MiR-125a-5p, miR-128-3p, miR-30d-5p results: ↑ Protection against MI/R injury, ↓ signaling of TRAF3, TRAF6, TNFRSF1B, BAK1, ↓ activation of caspases, MAPK pathway, ↓ apoptosis	Male mice; MI/R induced by coronary artery occlusion; 4-week exercise intervention, surgical BAT ablation, BAT miRNA inhibition

MiR, microRNA; BAT, brown adipose tissue; MI/R, myocardial ischemia/reperfusion injury; ROS, reactive oxygen species; MAPK, mitogen-activated protein kinase; TRAF3, TNF receptor-associated factor 3; TRAF6, TNF receptor-associated factor 6; TNFRSF1B, tumor necrosis factor receptor superfamily member 1B; ↑ increased, ↓ decreased.

## 3. Conclusions and Future Directions

**Figure 2:**
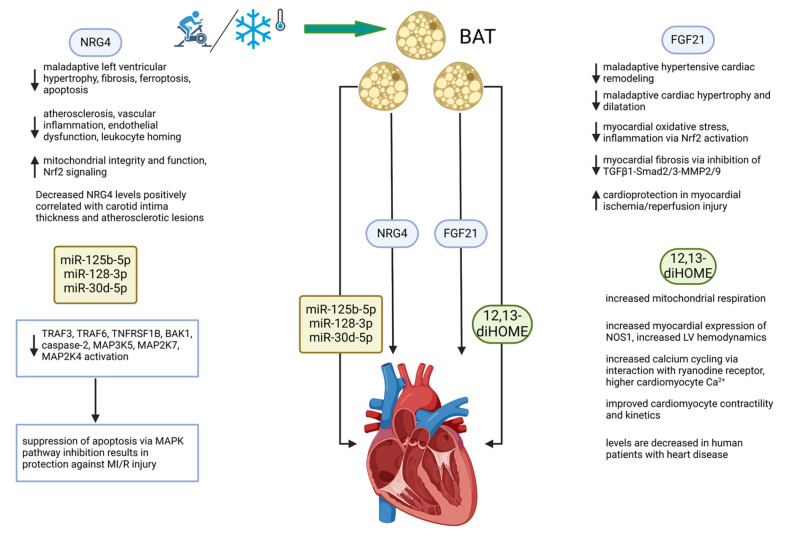
**BAT directly and favorably modifies cardiac function through the secretion of batokines.** The BAT secretome operates through at least four types of secreted molecules. These include the proteins FGF21 and NRG4, the lipokine 12,13-diHOME, and a set of miRNAs. Mechanisms of action include those that are indicated under the 4 factors diagrammed. Stimuli that activate BAT include exercise and cold exposure, both of which operate systemically and by direct targeting of multiple organ systems. See list of abbreviations for definitions of abbreviations used in the diagram. Created in BioRender.com. ↑ increased, ↓ decreased.

The discovery of functional BAT in humans entailed a profound shift in our understanding of adipose tissue in general as a multifunctional tissue, serving not only to store energy substrate but also as a thermogenic organ with endocrine, paracrine, and autocrine effects that play key roles in mediating inter-organ metabolic communication [5,66,88,89,90,91,92,93]. BAT has been shown over the last decade to act not just solely as a thermogenic organ but as an endocrine organ with pleiotropic effects in distant tissues that appear to be almost uniformly beneficial in all model systems studied thus far [28]. The mechanisms by which BAT communicates with distant tissues include proteins, lipokines, and exosomes, carrying a cargo of miRNAs that alter the metabolic functions of various organs, which, notably, include the heart [93]. Figure 2 provides a schematic outline of how activation of BAT and the four batokines highlighted in this review mechanistically affect cardiac function in a generally beneficial manner, thereby showing potential as cardioprotective factors.

This review has focused on elements of the BAT secretome which have been shown to affect the heart, specifically FGF21, neuregulin-4, 12,13-diHOME, and exosomes encapsulating miRNAs that exert cardiac effects. Given that CVD continues to be the primary cause of death globally, the urgency of discovering new preventative and therapeutic modalities for CVD is readily apparent [94]. BAT has received relatively little attention compared to other tissues with endocrine function in terms of its relevance as a cardioprotective organ, and it is notable that BAT is activated by exercise, an intervention with irrefutable beneficial effects on the cardiovascular system [66,92,93]. The recognition of BAT as an endocrine organ has stimulated recent investigation into its potential in promoting heart health, and the mechanisms by which BAT modulates cardiovascular function are only beginning to be elucidated. The data summarized in this review examine new mechanistic insights into how BAT activation and secretion of batokines favorably affect the heart.

BAT modulates cardiovascular function systemically by increasing EE and thereby acting in opposition to the development of obesity and clearing lipids from the circulation, which are two mechanisms by which ectopic lipid accumulation can be abrogated or at least minimized. However, the data from studies summarized in this review provide evidence of a direct line of communication between BAT and the heart, with various beneficial direct effects on the heart specifically and the cardiovascular system more generally. FGF21 specifically of BAT origin protects the heart from maladaptive remodeling in hypertension, myocardial ischemia/reperfusion injury, and acute myocardial infarction [31,41,42,43]. NRG4 has been shown to decrease cardiac fibrosis, apoptosis, ferroptosis, oxidative stress, and atherosclerosis, while also inducing protective adaptations including increased mitochondrial function and enhancement of anti-inflammatory activity via Nrf2 signaling [56,57,58,60,62]. 12,13-diHOME, a novel lipokine of the oxylipin class, exerts beneficial cardiovascular effects via enhanced calcium cycling with consequent improvements in systolic and diastolic function, with concomitant augmentation of cardiomyocyte shortening and kinetics [66,77]. MiRNA contained in small extracellular vesicles has also been shown to favorably modulate cardiac function. Specifically, miR-125b, miR-128-3p, and miR-30d-5p exhibited cardioprotective effects by inhibition of apoptosis in MI/R injury via suppression of the proapoptotic MAPK pathway [81]. While findings are divergent in terms of the effects of miR-92a on the cardiovascular system [83,84,85,87], one study found that in rats subjected to MI/R injury, overexpression of miR-92a resulted in improved systolic function and a reduction in infarct size [83].

While the findings of research on batokines and the heart available thus far are intriguing, limitations exist that, if addressed, would advance the field and allow for more definitive recommendations on specific interventions to be formulated. The mechanistic studies that constitute the main part of this review have utilized animal and cell culture models and therefore are preclinical, allowing only limited insight into how batokines and BAT expansion might beneficially affect cardiovascular function in humans. While our ability to investigate cardiac alterations in humans at the cellular level in vivo is ethically constrained, advanced imaging techniques can allow for functional changes in cardiovascular function to be assessed non-invasively. Demonstrations of advantageous changes in cardiovascular parameters in response to BAT activation and secretome function in human studies would provide exciting new data. Future mechanistic research should also include both females and males in rodent studies to clarify whether there are sex-specific differences in circulating batokines and responsiveness to batokines in a diverse range of tissues, including the heart [93]. It is reasonable to hypothesize that sexually dimorphic patterns of batokine activity might exist in humans, considering the evidence that young lean women have a higher percentage of BAT than age-matched young, lean men [95].

It is an exciting time to study BAT and its effects on cardiometabolic health; many questions remain surrounding the exact interventions utilizing expansion of BAT mass and batokine levels/activity, and the techniques available to disentangle the complex interactions between BAT and other organ systems are robustly suited to the task. Exercise and intermittent cold exposure are physiological means by which BAT mass can be expanded and it is reasonable to infer that expansion of BAT would lead to consequent increases in batokines. An interesting direction of future study would be to investigate the time course necessary to induce secretion of batokines in response to physiological stimuli such as cold exposure and exercise. Addressing this issue could allow for fine-tuning of interventions such as therapeutic cold exposure and the duration, time, and type of exercise necessary to promote batokine secretion. Modalities such as pharmacological adrenergic stimulation and direct exogenous delivery of BAT-derived secretory factors merit further exploration, although the risk of side effects and dosing issues must be carefully considered, along with the bioavailability and absorption characteristics of exogenous batokine delivery.

## Figures and Tables

**Table 1:** Summary of studies cited on FGF21’s cardiometabolic effects.

Year	First Author	Citation	FGF21 Effects on Cardiac Function (Systemic or Direct)	Model
2010	Hondares	[29]	↑ Hepatic FGF21 expression, ↑ thermogenic activation of neonatal brown fat	Swiss mice (fetal and postnatal), newborn + 3 months old, obesity induced by HFD (42% kcal fat)
2011	Hondares	[30]	↑ FGF21 expression and release in BAT, ↑ systemic protective effects	Swiss adult male mice + male Wistar rats (50–60 days old); cold exposure (4 °C for 6 h, 24 h, or 30 days)
2018	Ruan	[31]	↑ BAT-derived FGF21 protects against maladaptive cardiac remodeling in hypertension	C57BL/6J male mice (10–12 weeks); induced hypertension
2016	Fisher	[32]	↑ Glucose homeostasis, ↑ insulin sensitivity, ↓ systemic inflammation	General physiological studies
2013	Bookout	[33]	↑ Adaptive responses to fasting, ↑ insulin sensitivity, ↑ cognitive effects	Male C57BL/6J mice (10–12 weeks old), standard chow or ketogenic diet; FGF21 mini-pump infusion
2014	Laeger	[34]	↑ FGF21 signaling during protein restriction, ↑ endocrine adaptation to dietary changes	Male Sprague Dawley rats + C57BL/6 mice (10–12 weeks old); control diet, low-protein diet (5–10%), ketogenic diet (1.8% carbohydrate)
2024	Khan	[35]	↑ FGF21-driven metabolic adaptations, ↑ behavioral motivation changes in response to diets	Male C57BL/6J mice, Fgf21-KO, + KlbCamk2a (brain-specific KO); 10 days on control or low-protein diet (5%)
2013	Planavila	[41]	↓ Maladaptive cardiac hypertrophy and dilatation in FGF21 KO mice, reversal with exogenous FGF21	Fgf21 KO and Pparα-null male mice (neonatal and 4-month-old); cardiac hypertrophy induced by isoproterenol

BAT, brown adipose tissue; FGF21, fibroblast growth factor 21; HFD, high-fat diet; KO, knockout; ↑ increased, ↓ decreased.

## Data Availability

No new data were created or analyzed in this study.

## Abbreviations

12,13-diHOME	12,13-dihydroxy 9Z-octadecenoic acid
AMI	acute myocardial infarction
AMPK	adenosine 5′-monophosphate (AMP)-activated protein kinase
A_2A_R	adenosine 2a receptor
BAT	brown adipose tissue
Batokines	brown adipose tissue adipokine
CAD	coronary artery disease
CD36	cluster of differentiation 36
CIMT	carotid intima media thickness
CVD	cardiovascular disease
EE	energy expenditure
eWAT	epidydimal white adipose tissue
FATP1	fatty acid transport protein 1
FGF21	fibroblast growth factor 21
iBAT	interscapular brown adipose tissue
ICAM-1	intercellular adhesion molecule 1
L-NAME	N(ω)-nitr-L-arginine methyl ester
MetS	metabolic syndrome
MiR	micro-RNA
MI/R	myocardial ischemia/reperfusion
MKNK2	MAP kinase-interacting serine/threonine-protein kinase 2
NF-κB	nuclear factor kappa B
NOS	nitric oxide synthase
eNOS	endothelial nitric oxide synthase
iNOS	inducible nitric oxide synthase
MAPK	mitogen-activated protein kinase
MCP-1	monocyte chemotactic protein 1
Nrf2	nuclear factor erythroid 2-related factor
NRG4	neuregulin 4
PPC	perivascular progenitor cell
PPAR-γ	peroxisome proliferator activated receptor-gamma
ROS	reactive oxygen species
RyR	ryanodine receptor
sEV	small extracellular vesicle
SR	sarcoplasmic reticulum
TG	triacylglycerol
TNF-α	tumor necrosis factor-alpha
TNFRSF	tumor necrosis factor super family
TRAF	tumor necrosis factor receptor-associated factor
WAT	white adipose tissue
